# Treatise on Midwifery, Etc.

**Published:** 1858-03

**Authors:** 


ART. IV.—*A Theoretical and Practical Treatise on Midwifery, including the Diseases of Pregnancy and Parturition, and the Attentions Required by the Child from Birth to the Period of Weaning.* By P. CAZEAUX, Adjunct Professor in Faculty of Medicine, Paris. Second American Edition, translated from the Fifth French Edition, by W. R. BULLOCK, M.D. 8vo., pp. 751, with 140 illustrations. Lindsay & Blakiston: Philadelphia, 1857.

WE have wasted as much time as most men, but certainly have not to answer for having read all the books that have been written and published on the subject of midwifery. This prefatory statement is needed in order to let our readers understand that when we assert, as we do, that M. Cazeaux's book is the most complete *we* have ever seen upon the subject, we hold ourselves open to the correction of any of our brethren who may have read more thoroughly, and will apply their reading to the benevolent task of enlightening us.

The fact that this book has, in a short space of time, passed through five editions, gives it so entirely the stamp of public approbation that it would be almost useless to recommend to the attention of the profession what has been already so widely and justly appreciated.

M. Cazeaux's experience has been ample; his opportunities, owing, doubtless, to his great ability, such as are afforded to but few of us. "Where much is given much is expected," and certainly our author has, by the enlightened and diligent employment of his gifts, both natural and acquired, more than realized the very largest expectation that could have been cherished by his most ardent and interested admirers.

Some of our medical reviewers complain of custom, which requires that our modern obstetrical writers should make their books into encyclopædias, —that diseases of women and the hygiene of children should not have place in the same volume,—that he who can find a little on every subject in a single publication may read no other, and thus become a smatterer, but not a student. In reading M. Cazeaux's fifth edition we have no such fear; he is never superficial and careless: numerous as are his subjects, he sets to work upon them with the same indomitable energy, and never seems to consider one subject as worthy of greater care and reflection than another. We, ourselves, consider that most of the diseases of women take their most natural place between the same covers with a work on obstetrics, and certainly the hygiene of children as naturally succeeds; such a production as the science of cooking a beefsteak to perfection would follow the art of cutting it with skill.

So far we defend the custom of printing together the obstetrical treatise and the diseases of women, not omitting the hygiene of young children. But the objection to us, in this combination of subjects, is, it swells the volumes to such a degree that it becomes a formidable task to read, and a most voluminous one to review them. This feeling, no doubt, in part arises from our peculiar idiosyncrasy, and yet there is more than mere indolence in the objection.

The truth is, that the careful study and digestion of such a book as M. Cazeaux's could never make a superficial student fat; the real objection is, that when volumes are so large, the generality content themselves with looking at the title-page, reading the preface, looking over a few favorite subjects, such as hemorrhage, rupture of the uterus, etc., and then feel assured that they have mastered the contents of a great book!

It is this affectation of reading which chokes us up with smatterers,—not the careful perusal of such masses of well-considered truths and intellectual industry as the work before us contains.

M. Cazeaux divides his book into six parts. As usual, the first is devoted to the pelvis, external and internal; articulations, ligaments, etc. etc.; parts of generation, together with an account of the modifications undergone by the ovarian vesicles; with the concomitant changes of the uterus, the characteristic appearance and changes of the corpus luteum, necessarily including an excellent disquisition on the function of menstruation.

Part second includes simple uterine pregnancy. All the changes of the ovum; the old theory of the decidua, with the new theory proving that this so-called new formation is simply a thickening of the uterine mucous membrane; the fœtus and appendages; placenta, umbilical cord, etc.; functions of the child, and diameters of the head at full term.

Abnormal pregnancy takes its natural succession, together with lesions, accidents, and diseases, displacements, presentations, and phenomena too numerous even to name in the few pages allotted to a reviewer's agony.

Part third treats of labor in general; presentations and positions, physiological phenomena of parturition, together with the attentions necessary to the mother and child. This part contains very able remarks on face presentations, and much practical information which may be perused with advantage by practitioners as well as students.

Dyslocia is the heading of the fourth part. This is the most extensive division of the work, and contains a vast amount of excellent material, treating comprehensively and clearly of almost every species of difficulty belonging either to the mother's formations and organization, or the child's deformity or mal-position, which can possibly complicate a tedious labor. The articles on hemorrhage and puerperal convulsions are admirably written; that which treats of hemorrhage is, we think, by far the best on that subject that we have ever read, and we must beg to be excused if we occupy the doubtless valuable time of our readers for a short space by making some few extracts from it. The study of hemorrhage, its causes and efficient treatment, is one so fraught with interest to the practicing physician and accoucheur, that observations and opinions of a man of our author's vast experience and intelligence must be read with eagerness and respect by every one who has the sense and feeling to appreciate the vast and terrible responsibilities which are sometimes allotted to him.

Our author divides the symptoms of uterine hemorrhage into general and local. The general symptoms he describes as other writers have done, as chiefly consisting of sensations of fullness in the pelvic region, pains in the loins and thighs, bearing down, difficulty of micturition, etc. etc. These phenomena, which are characteristic of a local uterine congestion, are accompanied, as he observes, by symptoms of general plethora; that is to say, by pains in the head, vertigo, dimness of vision, flushing of the face, and by frequency and fullness of the pulse.

The local symptoms are divided into either external or internal. Of course we consider it as unnecessary to treat our readers to M. Cazeaux's definition of external and internal hemorrhage. When external, it will certainly speak for itself with sufficient clearness; but it is as certain that an internal flow of blood, particularly in the earlier months of gestation, might readily take place without detection, though, as M. Cazeaux says:—

"If the amount of blood should be considerable, the clot formed by the coagulation constitutes a foreign body, whose presence excites colicky gripings and pains in the loins, and a feeling of weight about the fundament; and these symptoms obstinately persist until a miscarriage takes place."

At an advanced stage of gestation, the writer observes that the hemorrhage may be so considerable as to produce a manifest enlargement of the abdomen, with, in some few cases, a well-marked fluctuation.

We confess that, under such circumstances, we should have little hope for either mother or child.

The doctor, on the subject of the seat of effusion, writes as follows:—

"The point at which the accumulation of blood takes place in these internal hemorrhages, that come on at an advanced period of gestation, must necessarily vary, according to the part of the utero-fœtal vascular apparatus which has been the source of the flooding;" (for instance,) "the blood may be effused primarily, between the uterine face of the placenta and the corresponding uterine wall; as the discharge progresses, it ordinarily dissects off the placenta toward some one point of its circumference, and is then effused all around the ovum by displacing the membranes. But it may also happen that the whole placental circumference remains adherent to the womb, while its central portion is entirely detached, the effusion being limited by the margins of this mass; the hemorrhage may be copious enough, in such instances, to kill the patient promptly.

"The blood may be effused into the tissue of the placenta, and constitute what has been called placental apoplexy. The woman's life is not compromised by such an accident; the death of the fœtus, and, as a consequence, its premature expulsion, would be almost a certain result."

Rupture of the umbilical cord is, of course, given as a cause of hemorrhage into the sac of the amnion. The author enters rather fully into the subject, and illustrates it by some interesting cases, which, in an article of this kind, we are compelled to omit. This rupture cannot affect the mother, but must, necessarily, be fatal to the child.

As this author denies the fact, so often repeated in our obstetrical books, that the neck of the womb begins to be, as it were, drawn out into its body at about the sixth month, and from that time gradually expands up to the period of gestation, at which time it has become the lower segment of the uterine ovoid, with only an aperture and ring to mark its location,—we say, as he positively denies all this, and maintains that the neck of the womb, though it gradually softens, does not begin its fusion into the lower segment of the organ until within a fortnight of full term, of course he cannot admit this development to be the cause, as hitherto supposed, of those frequently recurring hemorrhages which, in cases of placenta previa, take place during the three latter months of gestation. M. Cazeaux admits that these floodings do take place, but does not attempt to explain the why or the wherefore; the following passage is the nearest approach to an explanation that we can find:—

"The cervix uteri (considering the period of gestation) is usually thicker, softer, and more spongy, because the placenta, by becoming fixed over this point, determines there a more considerable afflux of blood."

The treatment of uterine hemorrhage (not unavoidable) has been so repeatedly discussed in books and lectures, that we cannot expect anything new upon the subject. M. Cazeaux tells us, as usual, to keep the woman quiet, use refrigerants, lay her on a hard bed, quiet her mind, and not to let her be made vexed or angry, if we can help it; in obstinate cases, when the flooding cannot be subdued by other means, recommends the administration of ergot, according to the advice of M. P. Dubois, whose opinion is that under such circumstances this medicine merely acts as a hemostatic, and does not produce uterine contraction unless uterine action has already commenced; in our opinion, a somewhat absurd position, for, if true, it would be perfectly useless in such a case. Indeed, the author himself seems to have an idea of its weakness, for he continues the subject after the following fashion:—

"Even supposing that it had this power, (the ergot,) that would not be a just ground of exclusion; for it must not be forgotten that the question before us is, that of arresting a serious accident—one which cannot continue without prejudice to both mother and child; and that the only other resource is the use of the tampon, which, even more than the ergot, would expose her to the hazard of a delivery before term."

All this is perfectly true, and renders the what we cannot help thinking over-nice distinction of the action of ergot somewhat frivolous; not that we deny M. P. Dubois's position, that ergot may have a hemostatic action apart from and independent of its peculiar action on the muscular contractility of the uterus; yet, at such a moment—for the author supposes that all other measures for arresting the flooding have been, to no purpose, tried—what, in the name of common sense, do we want but immediate and active contraction? for we maintain that at such a time the child's life is as a feather in the scale when weighed against the mother's; and no man should, in such a strait, attend either our wife, or daughter, or sister, who, to our knowledge, thought otherwise.

The author, in speaking of the special therapeutic measures to be used in flooding, says:—

"These vary, as stated, according to the abundance or trifling character of the discharge, and according to whether the latter is manifested in the course of the gestation or during labor. We shall first examine them during pregnancy."

Moderate hemorrhage, occurring in the last three months.

"If the flooding has been preceded by the general phenomena of plethora, and if, at the time the woman is examined, the pulse be found full, strong, and developed, the face flushed, etc.—in a word, if the hemorrhage appears to be owing to or kept up by the plenitude or morbid action of the vessels, it is necessary to have recourse to general venesection, which will act both as a revulsive and as an antiphlogistic; but this measure is recommended in those cases only in which labor has not yet commenced, and when the discharge is inconsiderable and has lasted but a short time. Blood-letting must be proscribed under the opposite circumstances, as also in those instances where the flooding is not associated with plethora.

"When the hemorrhage is not very abundant, and, as a consequence, when there is some reason to hope that the pregnancy will continue on its regular course, opiates may be administered; they might be given by the mouth, but it is much better, in general, to exhibit them by injection, in the dose of twenty drops of Sydenham's laudanum, diffused in a small quantity of some mucilaginous vehicle, and this may be repeated three or four times, at intervals of an hour or more, where the first has not been sufficient to arrest the symptoms."

A long experience, says Burns, enables me to recommend this measure in all cases where blood-letting is not practicable. Along with a strict regimen, such are the measures advised by M. Cazeaux, in cases of moderate hemorrhage, occurring in the last three months of gestation; he adds, they should be continued until it has entirely disappeared. Profuse hemorrhage occurring in the last three months—

"Where the flooding is more abundant the remedies to be employed are also more active; and to the measures already enumerated, except venesection, which, as before stated, must be rejected when the discharge is very profuse, we may now add," * * * *

"The application of compresses, steeped in some very cold liquid, to the upper part of the thighs, hypogastrium, or loins, (in one instance, M. Gendrin successfully administered an opiate injection at the temperature of melting ice,) and where the heat is very great, cold sponging over the legs, arms, and even the body. But the action of cold is not to be resorted to without discrimination, nor, as a general rule, should it be kept up for a very long time; because, although its application may be useful at the commencement of the attack, when the phenomena of local congestion are manifest, it would certainly prove injurious if a very copious and persistent hemorrhage had already enfeebled the patient, and if there was reason to fear the powers of life were giving way and that the woman was likely to sink into a state of complete prostration."

When the flooding cannot be arrested by other means, and the violence of the symptoms endangers the lives of both mother and child, the author, of course, recommends that the membranes should be ruptured, and admits the propriety of administering ergot.

M. Cazeaux speaks in high terms of the tampon, and gives minute directions as to the when and how it should be used. In cases of placenta previa, where the os uteri is too close to admit of turning, or the application of the forceps, he prefers its use to the employment of Dr. Simpson's suggestion—to detach the placenta completely from the internal surface of the uterus. In the course of the author's remarks on the distinguished Edinburgh professor, he says:—

"Professor Simpson has proposed to separate completely and bring away the placenta, whenever its insertion upon the neck has given rise to a hemorrhage which threatens the life of the mother; although rather too absolute at the outset, Mr. Simpson has finally yielded to the numerous and valid objections made to his precept, so far as to confine its application to the following conditions:—1. When the flooding has resisted the principal measures, and especially the rupture of the membranes and evacuation of the waters; 2. When the slight dilatation or development of the cervix, or contraction of the pelvis, render turning or any mode of artificial delivery dangerous or impossible; 3. When the death or immaturity of the fœtus restricts the duty of the accoucheur to caring for the safety of the mother. It is, therefore, especially with primiparous females, in cases of premature labor or rigidity of the cervix, and of its spasmodic contraction, of organic narrowing of the pelvis or of the genital passages, of the death or non-viability of the fœtus, and, finally, of extreme exhaustion of the mother, that the artificial separation may be practiced. It is to be understood, he adds, that in case of separation or extraction of the placenta, the fœtus should be withdrawn immediately, unless the hemorrhage should cease, which it does in the great majority of cases."

Even with this reservation, M. Cazeaux does not approve of Dr. Simpson's advice, but thinks that when the flooding continues after the evacuation of the waters, and when the neck does not allow the hand to be introduced, there is some chance left of saving both mother and child by applying the tampon.

With all respect for the opinion of our author, we cannot help thinking that the idea of saving the child's life under such circumstances is somewhat chimerical.

In the midst of this vexed question we enjoy one consolation, that is, that though the precise mode in which it is to be effected is a fruitful source of argument, yet all authorities agree that when the placenta presents, and the woman is in danger, she ought to be delivered as soon as possible. The truth is, the student must examine carefully every authority on the subject. When he has done so with all the ability and industry of his nature, he will still find, when the hour of trial comes, that his success in practice will mainly depend on his own judgment and sagacity in seizing the right moment for employing the means of relief he may determine on.

This article closes with a table by Professor Dubois, a summary of the treatment of hemorrhages affecting the latter months of pregnancy and during labor.

This vade mecum, as it is called, is an excellent thing for young gentlemen who are not addicted to study, and old gentlemen who have no time to read.

The subject of puerperal convulsions is a very interesting one, and its causes, predisposing and exciting, are ably discussed.

Investigation seems to have proved that the presence of albumen, if constant, in the urine of pregnant women, ought to excite our suspicions of the possibility of convulsions.

The author says:—

"Since albuminuria is present in the immense majority of eclamptic women, it, or rather the disease of which it is a symptom, may be rightfully regarded as the predisposing cause of eclamptic convulsions,—I say, the only known predisposing cause; for since attention has been fixed on this point, of all pregnant women those only who are affected with albuminuria (a few cases excepted) have been known to be attacked with convulsions."

Though all eclamptic patients have albuminuria, it is not insisted that albuminuria, however severe, necessarily gives rise to convulsions, for M. Cazeaux proceeds as follows:—

"Happily, it is by no means uncommon for pregnant women to have the urine highly charged with albumen, without presenting a single convulsive symptom. Of 41 women with albuminous urine, observed by M. Blot, but 7 had convulsions; and of 20 mentioned by MM. Devilliers and Regnault, 11 only were affected with them. The latter gentleman, to be sure, examined the urine of such women only as were dropsical; and it is very certain that many cases of albuminuria are not attended with infiltration. Still, by taking the mean between these different results, and having regard to my own observations, I think that I come near the truth in saying that one out of every four or five patients with albuminuria will be affected with convulsions."

We indulged our readers to the foregoing quotations for the purpose of drawing their attention to a most interesting subject, and regret that time and space prevent our entering upon it more fully.

Of course, no striking novelty can possibly present itself in the treatment of puerperal convulsions.

The remainder of this fourth part is devoted to rupture of the womb, together with the mechanical and surgical part of the obstetrical science.

The fifth division of the book treats of the delivery of the after-birth. The article is long and elaborate, but its merits will amply repay a careful perusal.

The sixth part, and "Last scene of all that ends this strange, eventful history," is not "Second childishness and mere oblivion," though it is devoted to nursing, lactation, weaning, and the hygiene of children.

In an appendix the author discusses the subject of anæsthetics, of the use of which he speaks in terms of approval; alludes to Dr. Simpson, of Edinburgh, as the first to venture on the use of anæsthetics in labor, and quotes his mode of administration.

The book is well translated, and reflects great credit on Dr. Bullock's intelligence and industry.

